# Some Account of the County Hospitals, and Other Charitable Institutions in Great Britain

**Published:** 1802-08-01

**Authors:** 


### *Some Account of the County Hospitals, and other charitable Institutions in Great Britain.*

THE methods of public and private Charity have their peculiar advantages; the fame benevolent principle directs both. Individual exertion is, however, but feeble when compared with the united efforts of many; and it is to thefe concentrated endeavours that England is indebted for her numerous public Inftitutions to relieve indigence and mifery, which beft proclaim the g ory and the praife of her national character. In the metropolis there is fcarcely a difeafe to which man is liable, fcarcely a calamity to which he is fubject, but it has an afylum open for its particular relief and confolation. To fhew that the fame fpirit of charity *characterises* the country, and has adorned inferior towns with the nobleft ornaments, (Hofpitals and Infirmaries for the fick and afflicted) is my prefent object. To thofe who, for want of reflection, do not calculate the many fources of human woe, an eftimate of the aggregate benefit of charities fo diffufed and extenfive may not be altogether unferviceable; and if it fhould have the good fortune to ftimulate th energies of thofe who have not yet felt the delight of doing good; to remind the gay and thoughtlefs, that there are other claims than thofe of diffipation upon their fuperfluity; and to encourage fimilar Inftitutions where none have yet been eftablifhed, the following account of Hofpitals, perhaps not generally known, will neither be confidered unneceffary nor unprofitable.

THE

THE COUNTY HOSPITAL AT YORK.

This Hofpital was inftituted in the year 1740, for the relief of the difeafed poor of the county of York; and the prefent edifice, which is a handfome ftone building, was erected as foon as the charitable fund would allow the expence. For many years it was the only inftitution of the kind north of the Trent, and in its infancy had many difficulties to ftruggle with; but through the attention, care and economy of its firft Patrons, efpecially of Marmaduke Fothergill, Efq. all obftacles to its progrefs were furmounted, and the great and extenfive utility of the defign procured for it fuch liberal affiftance, that though at firft the lower wards only could be opened, in a few years the upper wards were alfo fitted up for ufe, fo that there are now twenty-nine beds for men, and the fame number for women. The annual accounts of patients cured and relieved, 300 on an average, abundantly prove how well the liberality of the Benefactors to this Charity is beftowed and employed. Every perfon who is a Benefactor of £.20, or a Subfcriber of £.2 *per annum*, is a Governor, and privileged to recommend one out-patient, or one in-patient at a time. A Subfcriber of £.3 *per annum*, may recommend one in-patient, and one out-patient at one time. A Subfcriber of £.1 *per annum* is entitled to recommend one out-patient and no more.

Two of the Contributors, refident in York, vifit the Hofpital once a month, to enquire into the behaviour and conduct of the patients and fervants, and report their obfervations to the Committee. As the Governors are defirous that the management of the Hofpital fhould be as open to public infpection as poffible, any Subfcribers who refide in the country, are authorifed to become additional Houfe-Vifitors, whenever bufinefs or inclination may bring them to York. Perfons who meet with accidents, requiring the immediate aid of furgery, are received at any time of the day or night, without recommendation, according to the emergency of the cafe. The number of patients admitted, and difcharged cured, fince the eftablifhment in 1760, has been confiderably more than 26,300; and here an interefting thought fuggefts itfelf:—What muft have become of thefe but for an inftitution like this?—Would they not have funk; would they not have perifhed, under the *accumulated preffure* of difeafe and poverty?

THE GENERAL INFIRMARY AT NORTHAMPTON.

The Northampton Infirmary was firft erected in the year 1744, as a County Hofpital; the prefent extenfion of its beneficial influence, as a general Infirmary, only took place within thefe few years. The fick, lame, and poor of all counties, ties, who can obtain the recommendation of a Subfcriber, now find an afylum in this charitable Inftitution, where nothing is denied that can promote their comfort, and nothing with-held that can contribute to their recovery. Here the affluent are certain that their benefactions are directed in the moft efficacious manner to the benefit of the afflicted, and the indigent have the advantage of the moft excellent and experienced advice, together with proper accommodation, conftant attention, and wholefome and nutritious diet, comforts rarely to be met with in the habitations of poverty, but the want of which too often obftructs the means there offered for their relief. In cafes of accident or fudden calamity, no recommendation is required; the fufferers are received at any hour, day or night. All Subfcribers of two guineas or more *per annum*, or Benefactors of twenty guineas at one time for the ufe of the Infirmary, with the Treafurer, Gentlemen of the Faculty, &c. are Governors of the Charity, and privileged to recommend patients. Since the Infirmary was firft opened in 1744, upwards of 30,400 patients have been difcharged, cured; an ample proof of its utility to individuals, and of its importance to the community. Of the number cured, confiderably above 8000 have been admitted upon fudden misfortunes, and cafes of emergency; of thefe there are frequently 300 in a year.

THE GENERAL KENT AND CANTERBURY HOSPITAL.

This Hofpital was inftituted on the 26th of April, 1793, for the relief of the difeafed, fick, or lame, of the County of Kent. If any thing can add value to an eftablifhment of this defcription, it is the feafon when the undertaking commenced; and it is to the honour of the Benefactors of this Charity, that, under the preffure of times like thefe, and the peculiar circumftances of the late war, they remembered the caufe of the diftreffed, and confidered the claims of thofe who endured the fame misfortunes, and in the hour of ficknefs or difeafe, without any means of fupport or relief. This Inftitution has notwithftanding been begun, conducted and carried on, to the infinite advantage of the County; and it is fcarcely doubted but thofe gentlemen of Kent, who have not yet contributed their affiftance, will unite their exertions with thofe of its prefent Benefactors, to continue the aid it affords to the deftitute, which otherwife muft unhappily be withheld; for, according to the annual report, juft publifhed, fo numerous are the applications for admiffion, fo great the increafe of expence, in confequence of the prefent price of provifions, that, though the benefactions which, in the courfe of laft year, were confiderable, have been expended, there remains at the clofe of the annual annual account, barely a furplus. The managers are therefore at prefent altogether dependent on the continuance of that liberality, which, they add, they have not hitherto failed to experience. All Benefactors of twenty guineas and upwards, and all Subfcribers of two guineas or more annually, during payment, are Governors. Every annual Subfcriber of one guinea, or Benefactor of ten guineas, has a right to recommend two out-patients within the year. Every Subfcriber of two guineas, or Benefactor of twenty guineas, is privileged to recommend one in-patient, and two out-patients, or four out-patients. Every Subfcriber of three guineas, or Benefactor of thirty guineas, one in-patient and three out-patients, or fix out-patients. Every fubfcriber of five guineas, or Benefactor of fifty guineas, two in-patients and four out-patients, or ten out-patients within the year. No patient can be admitted but by the recommendation of a Subfcriber, Benefactor, or Deputy, unlefs in cafes which admit not of delay, (four beds being always referved for accidents,) when the Apothecary or Matron may receive patients, giving immediate notice to the Phyfician or Surgeon of the week.

It ought to be mentioned that the Phyficians, Surgeons, and fuperintending Apothecary, generoufly give their attendance gratis.

Among the Benefactors to this Hofpital for the laft year, (1800) we find the names of the Honourable George Watfon, Member for Canterbury, £. 100. Jofeph Boyle, Efq. (2d benefaction) 52*l.* 10*s.* and William Scott, Efq. (3d benefaction £. 25; of Mr. John Parnell, Mrs. Kingsford, and John Denne, Efq. twenty guineas each; and the Theatre liberally contributed 49*l.* 9*s.* the profits of a reprefentation. Since the Inftitution, 1083 in-patients and 1255 out patients have been admitted; of the former, 447 have been cured, 117 received material benefit, and 342 made out-patients; of the latter, 535 have been cured, 144 received benefit, and 245 made in-patients; 111 now remain in the Hofpital under cure. Total of in and out-patients admitted in the year 1800, was 552. Daily average of in and out patients in the fame year 106.

### THE GLOUCESTER INFIRMARY.

This Infirmary was founded in the year 1755, and endowed by its liberal Benefactors for the relief of the fick and lame of any county or nation, who may be deftitute of the means of fupport. That its advantages may be continued to patients of this defcription, Subfcribers are earneftly requefted to inform themfelves particularly with the actual circumftances of thofe they recommend, as fome are known to have been admitted, who who could have afforded the expences of ficknefs without inconvenience, whilft others have endeavoured to obtain a recommendation by making pecuniary offers to the Faculty for their attendance, provided they would not oppofe their admiffion into the Infirmary. To admit fuch perfons is to divert the ftream of benevolence from its intended courfe; to diminifh the power of relieving the really deftitute, and to promote the purpofes of private intereft or domeftic convenience; not without injuftice to the Faculty, who devote their time and fervices to the Charity, and have a right, either to the juft emoluments of their profeffion, or to the reflection that they are adminiftering to the relief of the *helpless* poor.

From the excellent rules for the government of this Infirmary we felect the following:

All Subfcribers of two guineas per annum, or Benefactors of twenty pounds or more, are Governors, and may attend and vote at all meetings of the Governors.

The Governors meet every Thurfday, at twelve o'clock, to admit or difcharge patients, and regulate the affairs of the Houfe.

Two Governors are appointed weekly to infpect and report the ftate of the Infirmary, and of the patients.

One of the Phyficians, with one of the Surgeons, attend at the Hofpital every Thurfday at eleven, to examine thofe who fhall be recommended for patients, and to receive under their care thofe who may be admitted. Every Wednefday and Saturday, at eleven o'clock, thefe alfo attend to prefcribe for the out-patients then on the book; and as often as particular cafes require.

The Apothecary, Secretary, and Matron conftantly refide in the Houfe, and fuperintend its economy in their refpective departments.

The perfons recommended for patients are admitted without expence; and when difcharged, are prefented with fome religious tract, and enjoined to go immediately to their houfe, and to return thanks in their refpective places of worfhip.

Every Subfcriber of one guinea is entitled to recommend two out-patients; of one guinea and a half, one in-patient; of two guineas, one in and one out-patient. A recommendation of an out-patient entitles fuch out-patient to continue on the books for the fpace of four months, when, if the out-patient fhould require further affiftance, a new recommendation muft be obtained.

Confiderably more than 21,000 in-patients have been under the care of this Infirmary fince its inftitution in 1755, of whom above 14,000 have been difcharged perfectly cured, independent of fome thoufands greatly relieved.

THE

THE RADCLIFFE INFIRMARY AT OXFORD.

This Infirmary is built of hewn ſtone, and upon a ſimilar plan with the Hoſpital at Glouceſter above-mentioned. An edifice of this deſcription had been long wanted at Oxford, as well for the immediate advantages of its foundation as for the benefit of the academical ſtudents in phyſic. It was erected by the Truſtees of Dr. Radcliffe's benefactions, out of the ſurplus money remaining after defraying the expence of his library. The ground was given by Thomas Rowney, Eſq. formerly high ſteward of the city, and the building was begun in May, 1759, and, being completed, fitted up and furniſhed, was opened for the reception of patients the 18th of October, 1770.

Like the Glouceſter Infirmary, this inſtitution does not limit its aſſiſtance to the unfortunate of a ſingle county, but affords an aſylum for the relief of the ſick and lame of all counties from whereſoever recommended. Every Subſcriber of one guinea per annum has the privilege of recommending one in-patient and one out-patient within every year; Subſcribers of two guineas per annum, can recommend two in-patients and two out-patients within the year; and ſo on in proportion, provided the in-patients do not exceed five in one year. No Benefactor of leſs than thirty guineas can recommend any patient. A benefactor of thirty guineas has the privilege of an annual Subſcriber of three guineas; and a Benefactor of fifty guineas has the privilege of an annual Subſcriber of five guineas.

No ſubſcriber's recommendation can be accepted whilſt his ſubſcription is in arrear: The Subſcribers to this charity are therefore requeſted to be early in making their payments, leſt the humanity of the acting Governors ſhould be put to the ſevere trial of refuſing admiſſion to miſerable objects for want of compliance in Subſcribers with the rules of the Hoſpital. In the courſe of the laſt year (1800) 618 patients were admitted into the Hoſpital, of whom 362 were diſcharged perfectly cured, beſides 96 greatly relieved, and 107 made out-patients. During the ſame period 284 out-patients were under the care of this Inſtitution, of whom 125 have been cured, and 76 ſo far relieved as to require little further aſſiſtance.

*** *Further communications relative to similar institutions in all parts of the kingdom will be acceptable to the Editors.*

*Observations*

